# Enhancing Crop Nitrogen Efficiency: The Role of Mixed Nitrate and Ammonium Supply in Plant Growth and Development

**DOI:** 10.3390/biology14050546

**Published:** 2025-05-14

**Authors:** Zhiqi Yang, Huifeng Yan, Haiwei Liu, Lan Yang, Guohua Mi, Peng Wang

**Affiliations:** 1 Key Laboratory of Tobacco Biology and Processing, Ministry of Agriculture and Rural Affairs, Tobacco Research Institute, Chinese Academy of Agricultural Sciences, Qingdao 266101, China; 2 College of Resources, Hunan Agricultural University, Changsha 410128, China; helloyanglan@163.com; 3 Department of Plant Nutrition, College of Resources and Environmental Sciences, China Agricultural University, Beijing 100193, China; miguohua@cau.edu.cn

**Keywords:** nitrogen efficiency, mixed nitrogen supply, plant growth, root architecture, carbon metabolism

## Abstract

Nitrogen fertilizers play a critical role in enhancing crop yields; however, excessive application has resulted in significant environmental challenges, including water contamination and increased greenhouse gas emissions. Therefore, improving nitrogen use efficiency is essential for sustainable agriculture. This review based on a systematic search of Web of Science and CNKI for peer-reviewed studies on maize nitrogen efficiency published between 1945 and 2024 (excluding conference abstracts), this review presents the first multiscale synthesis demonstrating how balanced nitrate–ammonium nutrition coordinates N–C metabolism and phytohormone signaling to boost nitrogen use efficiency and stimulate maize growth, with supporting evidence from other crops. By integrating results from hydroponic and field experiments, the review evaluates the influence of mixed nitrogen sources on nitrogen uptake, root morphology, photosynthesis, carbon metabolism, and hormone signaling. Findings indicate that optimal NO_3_^−^:NH_4^+^_ ratios improve nitrogen absorption through enhanced root development and activation of specific nitrogen transporters. Additionally, mixed nitrogen nutrition increases photosynthetic efficiency, promotes carbon assimilation, reduces energy expenditure, and stimulates auxin-mediated growth. This review shows that balanced nitrate–ammonium co-application synergistically enhances crop nitrogen-use efficiency and yield, provides a theoretical basis for high-efficiency nitrogen-fertilizer development, and helps alleviate environmental pressures, advance sustainable agriculture, and secure food and ecosystem safety. Its efficacy, however, is modulated by soil type, climate, and genotypic variation, necessitating systematic validation and application optimization in future research.

## Simple Summary

Excessive use of nitrogen fertilizers in agriculture has caused serious environmental problems, including water pollution and greenhouse gas emissions. Improving nitrogen use efficiency in crops is essential for sustainable agricultural development. This review discusses how a balanced mixture of nitrate (NO_3_^−^) and ammonium (NH_4_^+^) nitrogen can significantly enhance plant growth and nitrogen use efficiency. Using maize as a primary model, this review shows that such a supply improves root development, enhances nitrogen uptake and assimilation, boosts photosynthesis and carbon use, and promotes the synthesis of key growth hormones. These physiological improvements result in higher crop yields compared to using only nitrate or ammonium fertilizers. Understanding these processes will help scientists and farmers develop better fertilizer management strategies. These practices can optimize nitrogen use, improve crop productivity, and reduce environmental impacts. Consequently, adopting mixed nitrogen fertilizers can support agricultural sustainability, enhance food security, and protect ecosystems.

## 1. Introduction

Nitrogen (N) is vital for plant growth and development. Over the past 50 years, China’s grain production has quadrupled, with N fertilizer playing a key role in this increase [1]. However, excessive N fertilizer use has led to various environmental issues, including nitrate leaching, water eutrophication, acid rain, increased greenhouse gas emissions, and soil pH reduction. Early studies indicate that China’s nitrogen use efficiency is only 30–40%, with much of the nitrogen being fixed in the soil and eventually leaching into deep soil or volatilizing into the atmosphere [2]. Improving N fertilizer efficiency in high-yield, intensive agricultural systems are critical for both resource optimization and environmental sustainability. Researchers typically focus on two strategies to enhance crop N efficiency: developing nitrogen-efficient varieties and improving fertilization methods. N efficiency is categorized into nitrogen uptake efficiency (NUpE) and nitrogen use efficiency (NUtE) [3]. During the vegetative phase, N efficiency is primarily reflected in changes to crop growth, involving processes such as nitrogen uptake, assimilation, and carbon-nitrogen metabolism. In the reproductive phase, N efficiency is more closely linked to yield and grain protein content, involving not only the aforementioned physiological processes but also nitrogen transport and utilization [4]. Understanding the relationship between nitrogen supply-induced physiological changes and the underlying mechanisms is crucial for improving crop nitrogen efficiency.

Nitrate (NO_3_^−^) and ammonium (NH_4_^+^) are the primary inorganic nitrogen sources absorbed by plants, collectively accounting for over 70% of the total anions and cations taken up [5], though plants can also absorb organic nitrogen, e.g., urea. Recent studies have revealed that, compared to the sole supply of moderate NO_3_^−^ or NH_4_^+^, a balanced ratio of NO_3_^−^ and NH_4_^+^ enhances plant growth (Table 1). This beneficial effect, however, results from the interaction between NO_3_^−^ and NH_4_^+^, rather than the independent contribution of each. Research suggests that this interaction is linked to the source-sink dynamics in plants, and may also depend on factors such as plant species, genotype, growth stage, soil pH adaptability, and carbohydrate storage [6]. At present, several mechanisms have been proposed to explain why a mixed NO_3_^−^ and NH_4_^+^ supply promotes plant growth: (1) maintaining rhizosphere pH and enhancing root nitrogen uptake [7,8,9,10,11,12]; (2) reducing energy consumption, improving photosynthetic efficiency, and enhancing carbon synthesis [13,14,15]; (3) improving carbon use efficiency [16]; and (4) regulating hormone synthesis to influence leaf expansion and tillering [14,16]. This study explores the physiological mechanisms by which mixed NO_3_^−^ and NH_4_^+^ supply enhances plant growth, with maize as the primary subject, to provide theoretical insights into improving nitrogen efficiency and developing new fertilizer formulations. Unless stated otherwise, the nitrogen concentrations reviewed in this study are at medium supply levels.

## 2. Mixed NO_3_^−^ and NH_4_^+^ Improve Plant Nitrogen Efficiency by Enhancing Nitrogen Absorption

Root architecture plays a crucial role in nutrient and water absorption, serving as a key factor for high crop yield and efficient nutrient uptake [54,55,56,57,58]. The enhancement of nitrogen (N) uptake efficiency in roots typically occurs through two main mechanisms: (1) increasing the instantaneous nitrogen uptake rate per unit root surface area [59], and (2) maximizing the contact area between the root and the soil, referred to as a “powerful root system” [4,58,59]. These two characteristics are both complementary and essential for improving nitrogen uptake. Therefore, it is critical to explore the relationship between nitrogen uptake rates and root morphological changes under mixed nitrogen supply conditions.

### 2.1. NO_3_^−^ and NH_4_^+^ Uptake Under Mixed NO_3_^−^ and NH_4_^+^ Supply

With the continued study of plant nitrate (*NRTs/NPFs*) and ammonium (*AMTs*) transporters, the mechanisms underlying nitrate and ammonium absorption have been further elucidated. For instance, maize root transporters responsible for nitrate and ammonium uptake include low-affinity nitrate transporters, for instance, *ZmNRT1.1A-ZmNRT1.1D*, *ZmNRT1.2*, and *ZmNRT1.3*, high-affinity nitrate transporters like *ZmNRT2.1* and *ZmNRT2.2*, and high-affinity ammonium transporters including *ZmAMT1.1A*, *ZmAMT1.1B*, *ZmAMT1.3*, and *ZmAMT3.1* [60,61,62]. While most plants typically exhibit higher ammonium absorption rates than nitrate [6], excessive NH_4_^+^ uptake can lead to ammonia toxicity, causing rhizosphere acidification. This not only inhibits NH_4_^+^ absorption but also increases its outflow rate, resulting in greater energy expenditure on futile cycles. Compared to the sole supply of either nitrate or ammonium, an optimal ratio of both can further enhance nitrogen absorption in plants, as observed in species, including rice [63], maize [11,16], strawberry [13], tomato [64], wheat [65], and pepper [66], playing a crucial role in improving nitrogen use efficiency.

When plants simultaneously absorb nitrate and ammonium, an interaction between the two occurs, with ammonium supply often inhibiting nitrate uptake in roots, as observed in species, for instance, barley, rice, rapeseed, and *Populus euphratica* [67,68,69,70,71]. Earlier studies have suggested that ammonium-induced inhibition of nitrate uptake may be related to the downregulation of nitrate transporter gene expression, such as *NRT2.1*, in the roots [70,72]. This regulation may depend on the concentration of nitrogen or nitrogen metabolites, such as free ammonium and amino acids (glutamine, asparagine), in the roots [16,73,74]. Recent studies indicate that supplying maize with a 75/25 nitrate-to-ammonium ratio reduces nitrate uptake by only 10% relative to sole nitrate nutrition, yet yields significantly higher uptake rates than other mixed ratios (e.g., 25/75 or 50/50). This optimized proportion therefore confers a relative promotion of nitrate absorption under mixed nitrogen supply. Further investigation suggested that this promotion is associated with the upregulation of low-affinity nitrate transporter genes such as *ZmNRT1.1A-ZmNRT1.1C*, *ZmNRT1.2*, and *ZmNRT1.3*, while high-affinity transporters like *ZmNRT2.1* and *ZmNRT2.2* are not major contributors to this effect [12]. Concurrently, in some species, nitrate has been shown to enhance ammonium uptake, as in rice [63], poplar [71], rapeseed [69], and wheat [75]. It has been speculated that this promotion of ammonium absorption by nitrate is linked to the regulation of *AMT1.1* protein dephosphorylation [76], potassium channel activation [77], or aquaporin activation [78,79]. Recent studies in maize further revealed that, compared to sole ammonium supply, ammonium absorption rate increased under mixed nitrogen supply, likely due to the upregulation of *ZmAMT1.1A* expression [12].

### 2.2. Root Morphology Under Mixed NO_3_^−^ and NH_4_^+^ Supply

A well-structured root system is one of the key factors ensuring high crop yield and efficient nutrient utilization [59,80]. To optimize nutrient uptake efficiency, roots must adapt through their inherent plasticity in response to the spatiotemporal variability of soil nutrient availability.

Recent studies have introduced the concept of the “ideal root system architecture for maize” tailored to intensive agricultural systems, highlighting the significance of root size, lateral root length, and density [59,81]. As a key element of root architecture, primary root length serves as an indicator of root size and depth [57,82]. Longer primary roots improve the efficient uptake of water and nutrients from the soil and reduce nitrate leaching to deeper layers, thereby minimizing nitrogen loss and mitigating the environmental issues associated with eutrophication. Early studies in Arabidopsis demonstrated that increased ammonium supply typically inhibits primary root elongation, while moderate nitrate levels promote it. Both effects are mediated through the regulation of cell division and elongation, with the accumulation and distribution of auxin in the root tip playing a critical role in nitrate and ammonium-mediated signal transduction [83]. Recent reviews have provided detailed insights into the mechanisms by which nitrate and ammonium regulate primary root elongation [84,85,86]. A similar phenomenon has been observed in maize [17]. Under moderate nitrogen supply, ammonium inhibits primary root elongation compared to sole nitrate, with mixed nitrogen supply yielding intermediate effects. Thus, in both Arabidopsis and maize, the elongation of primary roots under mixed nitrate and ammonium supply is likely influenced by the independent effects of both nitrate and ammonium.

Lateral roots, as another key component of root architecture, play a crucial role in increasing the contact area between the plant root system and the soil, thereby enhancing water and nutrient uptake. From a carbon utilization perspective, changes in lateral root length and density are relatively economical and efficient, promoting optimal carbon use in the root system [57,59,81,87]. Early studies found that localized nitrate supply promoted lateral root elongation in maize [34], while both nitrate deficiency (<1 mM) and excess (>10 mM) in uniform solutions inhibited lateral root growth [88,89]. In Arabidopsis, increased ammonium supply typically inhibited lateral root elongation, while localized ammonium application stimulated lateral root branching along the primary root [90,91]. From early studies on ammonium and nitrate mediated lateral root development, it is clear that auxin plays a central regulatory role, a topic that has been thoroughly discussed in recent reviews [86]. Recent research in maize has shown that, compared to moderate nitrate supply, mixed nitrogen supply significantly increased lateral root density, with no significant difference compared to sole ammonium supply. This effect may be associated with higher auxin concentrations in the meristematic zone under mixed nitrogen supply, suggesting that this root morphology is more influenced by ammonium. Additionally, a more intriguing finding in maize showed that, compared to moderate nitrate or ammonium supply, mixed nitrogen supply consistently increased the average lateral root length per axis [17]. This result indicates an interaction effect between nitrate and ammonium, which enhances lateral root elongation.

## 3. Mixed NO_3_^−^ and NH_4_^+^ Improve N Availability by Regulating N Assimilation Rate

Nitrogen assimilation is a critical process by which plants convert inorganic nitrogen into organic forms, directly influencing plant growth and development [12]. Compared to sole nitrate supply, mixed nitrogen supply significantly enhanced nitrate reductase (NR) activity in maize roots, with no notable effect on NR activity in the shoots. In contrast, NR activity in both roots and shoots was markedly suppressed under sole ammonium supply [12,17]. This phenomenon may be explained by earlier studies, which suggest that mixed nitrogen supply reduces nitrate reductase protein (NRP) levels while enhancing nitrate reductase activity (NRA) compared to sole nitrate supply [92]. However, both NRP and NRA are inhibited under sole ammonium supply. In the maize growth experiments, although the exogenous application of the NR inhibitor Na_2_WO_4_ suppressed above-ground growth under both mixed nitrogen and sole nitrate supply conditions, the above-ground biomass was significantly higher under mixed nitrogen supply compared to sole nitrate supply. This suggests that, despite the inhibition of NR activity, mixed nitrogen supply can still promote maize growth to some extent. This could be due to enhanced nitrogen absorption and utilization efficiency under mixed nitrogen conditions, or the activation of compensatory mechanisms that contribute to improved growth performance [12,17].

Recent studies have shown that changes in glutamine synthetase (GS) activity within plants are crucial for mediating plant growth under mixed nitrogen supply conditions. Research in maize has shown that, compared to sole nitrate supply, mixed nitrogen supply significantly increases GS enzyme activity in both above-ground tissues and roots. Although enzyme activity may be lower than that under sole ammonium treatment, it promotes nitrogen assimilation rates and amino acid synthesis [12,17]. The balance between free ammonium and amino acid concentrations mediated by GS under mixed nitrogen supply is a key factor driving plant growth. For instance, in maize, under a 1 mM nitrogen supply, mixed nitrogen supply maintained or slightly increased ammonium concentration in the above-ground tissues compared to sole nitrate supply, while sole ammonium supply increased ammonium concentration in the above-ground tissues by 2–3 times. Root ammonium concentration slightly increased under mixed nitrogen supply, whereas sole ammonium supply significantly elevated it [12,16,17]. Additionally, compared to sole nitrate supply, mixed nitrogen supply left Gln concentration unchanged in the above-ground tissues, while Asn increased by 4.02 times. In the roots, Glutamine (Gln) and Asparagine (Asn) concentrations increased by 1.77 and 4.09 times, respectively. Under sole ammonium supply, Gln concentration in the above-ground tissues increased by 1.64 times, Asn by 18.64 times, and root Gln and Asn by 3.34 and 32.29 times, respectively [16]. Preliminary evidence suggests that, compared to sole nitrate supply, mixed nitrogen supply enhances above-ground biomass and leaf area, with GS playing a pivotal regulatory role. Ammonium assimilation under mixed nitrogen supply reduces energy consumption while increasing GS activity in the roots, promoting the conversion of ammonium to Gln and improving nitrogen assimilation efficiency. This process also prevents the excessive accumulation of endogenous ammonium, as seen with sole ammonium supply, which can inhibit growth and leaf expansion, or the buildup of Gln and Asn, which reduces energy utilization efficiency.

## 4. Mixed Supply of NO_3_^−^ and NH_4_^+^ Enhances Carbon Availability by Promoting Carbon Synthesis and Metabolism

### 4.1. Photosynthetic Rate

The form of nitrogen supplied to plants can influence their carbon source activity (photosynthetic efficiency), which may contribute to growth differences [16,46]. The primary factor underlying these differences is energy utilization. Assimilating 1 mole of nitrate requires 8 moles of electrons and 16 moles of ATP, in contrast to ammonium assimilation, with the majority of these equivalents derived from photosynthesis, particularly ATP produced via photophosphorylation [93]. Research indicates that the reductant required for nitrate reduction in leaves is 2.5 times the assimilate needed for CO_2_ fixation into carbohydrates [94]. Consequently, nitrate assimilation can impact photosynthetic rates, especially under specific conditions including low light intensity, high-density planting, inadequate or excessive CO_2_, and during periods of active reproductive growth [11,46]. For example, under low light, nitrate treatment alone reduces photosynthetic rates in species like Chinese cabbage, wheat, and tomatoes, whereas mixed nitrogen supply maintains higher photosynthetic efficiency [46,95,96,97]. In high-density maize planting, both mixed nitrogen and ammonium alone significantly increase photosynthetic rates compared to nitrate alone, with no significant difference between the latter two [16].

Ammonium-based nutrition enhances the photosynthetic process in plants through two primary mechanisms, in addition to energy conservation: (1) It modulates leaf stomatal conductance, thereby regulating CO_2_ supply. Ammonium nutrition, compared to nitrate alone, increases stomatal conductance in white clover and Chinese cabbage, thereby boosting photosynthetic rates [46,98]. (2) It enhances thylakoid electron transport (light reaction) by increasing chloroplast volume, mesophyll cell conductivity, photosynthetic electron transport rate (ETR), and the maximum photochemical quantum yield of PSII (Fv/Fm) [46,99]. This effect is likely attributed to increased content or activity of photosynthetic electron carrier proteins [26,46]. Additionally, compared to nitrate alone, ammonium nutrition upregulates the activity of Rubisco, glyceraldehyde-3-phosphate dehydrogenase (GAPDH), fructose-bisphosphate aldolase (FBA), fructose-1,6-bisphosphatase (FBPase), and TK kinase, while also enhancing the expression of related genes in Chinese cabbage [46]. Ammonium nutrition similarly increases Rubisco content in the leaves of beetroot and rice [26,99].

While ammonium-based nutrition can enhance plant photosynthetic efficiency to some extent, does a higher ammonium supply always result in improved photosynthesis? Studies indicate that, compared to nitrate alone, ammonium nutrition can increase Ribulose-1,5-bisphosphate (RubP) regeneration capacity in plants. However, excessive RubP production can lower intercellular CO_2_ concentration in leaves, promoting photorespiration [26]. Early research found that under certain ammonium supply conditions, photorespiration rates were 20 times higher than with nitrate [100]. The accumulation of ammonium ions in leaves disrupts photophosphorylation in chloroplasts, inhibiting photosynthesis [101]. Sorghum studies demonstrated that when ammonium concentration reached 5 mM, stress symptoms emerged, leading to reduced growth and photosynthesis [102]. In rice, high light intensity combined with ammonium supply showed a negative interaction, likely due to increased ammonium transport to the leaves, which impaired specific photosynthetic processes [103]. For instance, compared to nitrate alone, excessively high ammonium supply inhibited net photosynthetic rate in spinach.

### 4.2. Carbohydrate Metabolism

Nitrogen assimilation requires carbon skeletons provided by the plant. Ammonium assimilation occurs at a higher rate than nitrate, promoting the rapid synthesis of amino acids (Gln and Asn) in plants [104,105,106,107]. α-Ketoglutarate (2-OG) serves as a key carbon skeleton in this process [108]. In Arabidopsis, ammonium nutrition enhances glycolysis and the tricarboxylic acid (TCA) cycle compared to nitrate, facilitating the synthesis of 2-OG, malate, and fumarate, which provide carbon skeletons for nitrogen assimilation [106,107]. In addition to 2-OG, oxaloacetate (OAA) is another crucial carbon skeleton in nitrogen metabolism [108]. OAA is synthesized via two pathways: one through the TCA cycle from malate, and the other via the PEP-CO_2_ pathway catalyzed by PEP carboxylase (PEPC), with the latter dominating nitrogen assimilation [104]. Studies in Arabidopsis and rice have shown that PEPC deficiency impairs OAA synthesis and ammonium assimilation [109,110], indicating that PEPC-mediated carbon flow is pivotal for ammonium assimilation, a conclusion also supported by research in maize [16]. In maize, PEP plays a central role in carbon metabolism, directing carbon flow. Under mixed nitrogen supply, PEP content in maize shoots increases compared to nitrate or ammonium alone. This enhances nitrogen assimilation by facilitating OAA synthesis through both PEPC and the TCA cycle. Additionally, PEP promotes tryptophan (Trp) synthesis via the shikimate pathway, stimulating auxin production. In contrast, under ammonium-only conditions, PEP content remains unchanged, and the high synthesis of Gln and Asn diverts significant carbon toward nitrogen assimilation, reducing both carbon and nitrogen utilization efficiency, and limiting carbon flow to the shikimate pathway, ultimately lowering auxin concentrations.

Sugars are important indicators of carbon (C) levels and utilization efficiency in plants, as well as signaling molecules [111]. The assimilation, transport, utilization, and storage of sugars are key indicators of plant source activity and carbon pools [112,113]. Under high nitrogen supply, higher sugar accumulation in leaves promotes leaf development and photosynthesis, whereas under low nitrogen conditions, gene expression related to photosynthetic tissue development may be suppressed [114,115]. Compared to nitrate alone, ammonium nutrition significantly increases net photosynthetic rates in winter wheat, maize, and Chinese cabbage, as well as glucose, fructose, and sucrose content in leaves, particularly under mixed nitrogen supply [16,46,116]. In maize, research shows that, compared to nitrate alone, ammonium nutrition enhances glucose, fructose, and sucrose levels in both shoots and roots, indicating improved carbon source activity. However, excessive ammonium supply promotes starch synthesis in plants, leading to a decrease in carbon utilization efficiency. Furthermore, starch, as a major carbon reserve in plants, is more readily converted under ammonium nutrition compared to nitrate supply [6]. Excessive starch accumulation may reduce carbon utilization efficiency and damage chloroplast function, such as limiting CO_2_ diffusion or impairing chloroplast integrity [117].

Trehalose-6-phosphate (T6P) plays a pivotal role in regulating plant metabolism, functioning as both a signal and a feedback regulator of sucrose levels by modulating carbon and nitrogen processes [118,119]. Early studies demonstrated that chemically increasing T6P levels in plants significantly improved wheat yield by regulating sugar distribution and utilization [118,120] found that elevated T6P levels stimulate nitrogen assimilation and promote starch synthesis, potentially enhancing starch production [118,119]. In maize, research showed that ammonium nutrition, when combined with nitrate, increases root T6P concentrations, which may act as a signaling molecule to induce Gln and Asn synthesis [16]. Further studies indicated that, compared to nitrate alone, ammonium nutrition alone increased root T6P and starch concentrations by 4.5 and 1.23 times, respectively. Under mixed nitrogen supply, root T6P increased by 1.87 times, while starch levels remained similar to those in nitrate-only treatments. These findings suggest that increased ammonium supply may elevate T6P levels, promoting starch synthesis and directing more sucrose from shoots to roots, thereby increasing Asn and Gln levels. The balance of these metabolites reflects the efficiency of carbon and nitrogen utilization in plants. Thus, appropriately increasing endogenous T6P levels can enhance carbon and nitrogen utilization, whereas excessively high levels may cause inhibitory effects.

## 5. Phytohormone Synthesis and Signaling Mediated by Interaction of NO_3_^−^ and NH_4_^+^ Supply

**Figure 1:**
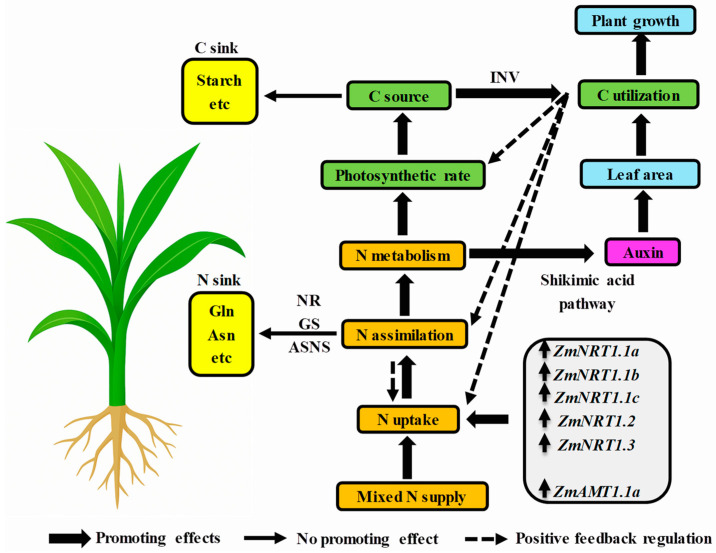
Exploring the potential mechanisms of growth mediated by mixed nitrogen supply: insights from maize as a model crop.

Indole-3-acetic acid (IAA) is a key endogenous hormone in plants, crucial for root growth and development [121,122]. Nitrogen regulates plant growth indirectly by influencing auxin synthesis, metabolism, and signaling pathways [122]. Auxin controls leaf development through leaf primordium initiation [123], vascular development [124] and leaf cell division and expansion [125]. The regulation of auxin synthesis and transport by varying nitrate concentrations has been documented in species, e.g., turnip cabbage [126], maize [127], soybean [128], pineapple [129], and Arabidopsis [130]. Studies show that nitrate, compared to ammonium alone, enhances auxin transport in roots and promotes root growth [131]. Nitrate directly regulates lateral root development and auxin synthesis and transport [132]. In tobacco, ammonium-only conditions result in lower auxin concentrations in the shoots compared to nitrate [26]. Interestingly, mixed nitrogen supply in tomato may increase auxin concentrations in the shoots compared to nitrate alone, potentially due to genotype differences [133]. A study on lettuce [134] showed that ammonium-nitrate nutrition increased IAA content compared to nitrate-only nutrition. In rice, a 75:25 ammonium-nitrate mixture was more favorable for IAA accumulation in roots and lateral root formation than ammonium alone [135]. Similarly, increasing NO_3_^−^ supply on an ammonium basis significantly boosted auxin levels in the shoots, phloem, and roots, indicating that ammonium-nitrate nutrition enhances auxin synthesis and its polar transport to roots [136]. Wang [16] demonstrated that mixed nitrogen supply in maize enhances Trp-dependent IAA synthesis via the shikimate and tryptophan (Trp) pathways, boosting the levels of phosphoenolpyruvate (PEP) and Trp, key precursors for IAA production. Transcriptome analysis further revealed significant upregulation of crucial genes involved in IAA biosynthesis, including DAHP synthase, indole-3-glycerol phosphate synthase, and YUCCA monooxygenases, under mixed nitrogen conditions. This resulted in increased IAA accumulation and activation of the auxin response pathway, ultimately promoting leaf growth. The findings of this study establish the relationship between nitrogen uptake, assimilation, carbon synthesis and assimilation, and hormone synthesis in maize (as shown in Figure 1). This relationship provides a systematic theoretical foundation for a deeper understanding of the role of mixed nitrogen supply in maize growth and the improvement of nitrogen use efficiency (Figure 1).

The data in this figure is sourced from published references [11,12,16,17]. Compared to sole nitrate or ammonium supply, mixed nitrogen significantly enhances nitrogen uptake in maize, promoting nitrogen assimilation and metabolism. This metabolic enhancement not only increases photosynthetic efficiency, thereby boosting carbon source activity and carbon utilization, but also regulates leaf area through auxin synthesis via the shikimate pathway, ultimately supporting plant growth. A series of low-affinity nitrate transporters (NRTs) likely facilitate nitrate uptake, while *ZmAMT1.1a* is likely involved in ammonium absorption. Although sole ammonium supply also promotes nitrogen uptake and assimilation, it leads to excess nitrogen storage, primarily as glutamine (Gln) and asparagine (Asn), resulting in nitrogen redundancy. Furthermore, under sole ammonium conditions, a significant portion of carbon is stored as starch, which reduces carbon utilization efficiency and thus hinders plant growth. Note: Thick arrows represent promoting effects, thin arrows indicate no promoting effect, and dotted arrows represent positive feedback regulation.

In addition to auxins, other hormones may play a role in regulating plant growth under mixed nitrogen supply. Walch [137] found that nitrate maintains a physiological balance of cytokinins in both shoots and roots, facilitating their transfer from roots to shoots and influencing morphogenesis, whereas ammonium suppresses cytokinin synthesis in the roots. However, recent maize studies report no significant differences in cytokinin levels or leaf area between ammonium-only and nitrate-only treatments. Under mixed nitrogen supply, plants exhibit the largest leaf area but the lowest cytokinin concentrations [16]. Furthermore, ABA, primarily synthesized in the shoots, induces stomatal closure, with ammonium supply promoting this process [138]. Conversely, in lettuce, an optimal ammonium-to-nitrate ratio in the mixed nitrogen supply reduces ABA content in leaves [134].

## 6. Conclusions and Prospect

The mixed supply of NO_3_^−^ and ammonium NH_4_^+^ enhances crop nitrogen use efficiency by mediating the dynamic interplay among plant nutritional demands, ecological processes, and anthropogenic inputs [139,140]. This review provides a comprehensive analysis of the mechanisms underlying mixed nitrogen supply (nitrate-ammonium combination) in maize and other crops, highlighting its profound impact on plant growth, nitrogen uptake and assimilation, photosynthesis, and carbon metabolism. The mixed nitrogen supply enhances nitrogen source activity in plants, thereby promoting nitrogen absorption and assimilation, which subsequently optimizes carbon metabolism and photosynthetic efficiency. Moreover, it supports the coordinated growth of both shoot and root systems via auxin synthesis and distribution, significantly improving nitrogen and carbon utilization efficiency and underscoring the intricate interaction between the plant’s nitrogen and carbon resource pools. Future research should prioritize the identification of nitrogen-use efficient genes and molecular breeding strategies to further dissect the molecular mechanisms governing mixed nitrogen supply in plant metabolism, ultimately enhancing crop nitrogen efficiency. Building on these foundational insights, practical applications should be explored, such as the development of advanced nitrogen fertilizers and the integration of precision agriculture technologies, to drive efficient and sustainable agricultural practices.

## Figures and Tables

**Table 1:** Optimum ammonium and nitrate ratios for growth of different plant species.

Species	Methods of Cultivation	Nitrogen Supply Concentration	Optimal Ammonium-Nitrate Ratio	Medium pH	Reference
Maize	Hydroponics	1 mmol L^−1^	1:3	5.8	[16]
	Hydroponics	1 mmol L^−1^	1:3	5.8	[11]
	Hydroponics	1 mmol L^−1^	1:3	5.8	[12]
	Hydroponics	1 mmol L^−1^	1:3	5.8	[17]
	Hydroponics	7.14 mmol L^−1^	3:1	5.3 ± 0.5	[18]
	Field	240 kg hm^−2^	1:3–1:1	--	[19]
	Field	180 kg hm^−2^	1:3	--	[20]
	Hydroponics	3 mmol L^−1^	1:4	4.8–5.5	[21]
	Hydroponics	11 mmol L^−1^	0:11–2:9	--	[22]
Wheat	Hydroponics	0.2 mmol L^−1^	1:5	5.6 ± 0.2	[7]
	Hydroponics	7.14 mmol L^−1^	1:3–1:1	6.0 ± 0.5	[23]
	Field	--	2:1	--	[6]
Barly	Hydroponics	7.14 mmol L^−1^	1:3–1:1	6.0	[24]
Rice	Hydroponics	2.86 mmol L^−1^	1:1	6.0	[25]
	Hydroponics	2.86 mmol L^−1^	1:1	5.50 ± 0.05	[26]
	Hydroponics	2.86 mmol L^−1^	1:1	6.0	[27]
	Hydroponics	2.86 mmol L^−1^	1:1	6.0	[28]
Soybean	Hydroponics	3 mmol L^−1^	0:1–1:4	6.4	[29]
	Hydroponics	2.4 mmol L^−1^	1:3	5.8	[30]
	Hydroponics	11 mmol L^−1^	1:10	--	[22]
*Phaseolus vulgaris*	pot	16 mmol L^−1^	1:3	--	[31]
Sorghum	Hydroponics	14.3 mmol L^−1^	1:4	--	[32]
Tobacco	Field	105 kg hm^−2^	1:1	6.21	[33]
	Hydroponics	10 mmol L^−1^	0:1–3:7	5.8 ± 0.1	[34]
	Hydroponics	2 mmol L^−1^	1:1	6.8–7.2	[35]
	Hydroponics	20 mmol L^−1^	1:1	6.9–7.1	[36]
Strawberry	Hydroponics	14.2 mmol L^−1^	1:1	6.5–6.8	[37]
	Hydroponics	7 mmol L^−1^	3:11	6.0	[38]
	Hydroponics	14.2 mmol L^−1^	1:3	6.5	[13]
Potato	Hydroponics	6.4 mmol L^−1^	1:1	5.5–6.5	[39]
	Hydroponics	41 mmol L^−1^	1:3	--	[40]
Tomato	Hydroponics	5 mmol L^−1^	1:3	6.2	[41]
	Hydroponics	3.83 mmol L^−1^	3:1	6.0 ± 0.2	[42]
	Hydroponics	12.5 mmol L^−1^	1:4	6.0	[43]
	Hydroponics	8 mmol L^−1^	1:3	5.5–6.5	[44]
	Hydroponics	14 mmol L^−1^	2:12	5.5–6.0	[45]
Cabbage	Hydroponics	5 mmol L^−1^	15:85	6.5–7.0	[46]
	Hydroponics	11 mmol L^−1^	1:10	--	[22]
	Hydroponics	--	1:3	--	[47]
Cucumber	Hydroponics	12.5 mmol L^−1^	11:3	--	[48]
Spinach	Hydroponics	12 mmol L^−1^	0:1–1:3	6.5	[49]
	Hydroponics	8 mmol L^−1^	1:1	5.5–6.0	[50]
Grape	Hydroponics	7.14 mmol L^−1^	3:7	6.5	[51]
	Field	Nitrogen 10 g plant^−1^	1:1	--	[52]
Sesame	Hydroponics	8 mmol L^−1^	1:9	5.85	[10]
Onion	Hydroponics	--	1:3	--	[53]

## Data Availability

All data generated or analyzed during this study are included in this published article.

## References

[1] Jiao X.Q., Lyu Y., Wu X., Li H., Cheng L., Zhang C., Yuan L., Jiang R., Jiang B., Rengel Z. (2016). Grain production versus resource and environmental costs: Towards increasing sustainability of nutrient use in China. J. Exp. Bot..

[2] Zhang H., Forde B.G. (2000). Regulation of Arabidopsis root development by nitrate availability. J. Exp. Bot..

[3] Moll R.H., Kamprath E.J., Jackson W.A. (1982). Analysis and interpretation of factors which contribute to efficiency of nitrogen utilization. Agron. J..

[4] Mi G., Chen F., Zhang F. (2012). Physiological Basis and Genetic Improvement of Crop Nutrient Efficiency.

[5] Caicedo J., von Wirén N., Arce O., Gijzen H.J. (2000). Effect of total ammonia nitrogen concentration and pH on growth rates of duckweed (*Spirodela polyrrhiza*). Water Res..

[6] Li S., Wang Z., Stewart B.A. (2013). Responses of crop plants to ammonium and nitrate N. Adv. Agron..

[7] Cox W.J., Reisenauer H.M. (1973). Growth and ion uptake by wheat supplied nitrogen as nitrate, or ammonium, or both. Plant Soil..

[8] Liu N., Zhang L., Meng X., Neelam A., Yang J., Zhang M. (2014). Effect of nitrate/ammonium ratios on growth, root morphology and nutrient elements uptake of watermelon (*Citrullus lanatus*) Seedlings. J. Plant Nutr..

[9] George J., Holtham L., Sabermanesh K., Heuer S., Tester M., Plett D., Garnett T. (2016). Small amounts of ammonium (NH_4_^+^) can increase growth of maize (*Zea mays* L.). J. Plant Nutr Soil Sci..

[10] Xu Z., Qin L., Shui Y., Han P., Liao X., Hu X., Xie L., Yu C., Zhang X., Liao X. (2017). Effects of different nitrogen form and ratio on growth and nutrient uptake of different sesame cultivars. Chin. J. Oil Crop Sci..

[11] Wang P., Wang Z., Sun X., Mu X., Chen F., Yuan L., Mi G. (2019). Interaction effect of nitrogen form and planting density on plant growth and nutrient uptake in maize seedlings. J. Integr. Agric..

[12] Wang P., Wang C., Wang X., Wu Y., Zhang Y., Sun Y., Shi Y., Mi G. (2023). Increasing nitrogen absorption and assimilation ability under mixed NO_3_^−^ and NH_4_^+^ supply is a driver to promote growth of maize seedlings. J. Integr. Agric..

[13] Wu Y., Xu Y., Liu Z., Cai M., Pan H., Zhang Q. (2024). Different responses of *Lagerstroemia indica* to varied supplies of ammonium and nitrate. Sci. Hortic..

[14] Miao Y., Li S., Fu Y., Wang Z., Xu X., Luo L. (2014). Characteristics of ammonium N and nitrate N accumulation in dryland soil in relation with wheat yield. Chin. J. Appl. Ecol..

[15] Zhu Z., Yu M., Chen Y., Guo Q., Zhang L., Shi H., Liu L. (2014). Effects of ammonium to nitrate ratio on growth, nitrogen metabolism, photosynthetic efficiency and bioactive phytochemical production of *Prunella vulgaris*. Pharm. Biol..

[16] Wang P., Wang Z., Pan Q., Sun X., Chen H., Chen F., Yuan L., Mi G. (2019). Increased biomass accumulation in maize grown in mixed nitrogen supply is mediated by auxin synthesis. J. Exp. Bot..

[17] Wang P., Yang L., Sun X., Shi W., Dong R., Wu Y., Mi G. (2024). Lateral root elongation in maize is related to auxin synthesis and transportation mediated by N metabolism under a mixed NO_3_^−^and NH_4_^+^ supply. J. Integr. Agric..

[18] Schrader L.E., Domska D., Jung P.E., Peterson L.A. (1972). Uptake and assimilation of ammonium-N and nitrate-N and their influence on the growth of corn (*Zea mays* L.). Agron. J..

[19] Peng Z.D. (2018). Effects of Different Nitrogen Forms and Mulching Methods on Nitrogen Metabolism and Yield Formation in Spring Maize.

[20] Li C.C. (2017). Regulatory Effects of Nitrogen Form Ratios on Nitrogen Utilization and Yield in Maize Under Full Film Double-Ridge Sowing.

[21] Schortemeyer M., Feil B. (1996). Root morphology of maize under homogeneous or spatially separated supply of ammonium and nitrate at three concentration ratios. J. Plant Nutr..

[22] Mohamed E., Wilcox G.E. (1990). Plant species response to ammonium-nitrate concentration ratios. J. Plant Nutr..

[23] Gashaw L., Mugwira L. (1981). Ammonium-N and nitrate-N effects on the growth and mineral compositions of triticale, wheat, and rye 1. Agron. J..

[24] Ali A., Tucker T.C., Thompson T.L., Salim M. (2001). Effects of salinity and mixed ammonium and nitrate nutrition on the growth and nitrogen utilization of barley. J. Agron. Crop Sci..

[25] Duan Y., Zhang Y., Shen Q. (2005). Effects of increased nitrate nutrition on ammonium uptake and growth of rice seedlings of different genotypes. Acta Pedol. Sin..

[26] Guo S., Zhou Y., Shen Q., Zhang F. (2007). Effect of ammonium and nitrate nutrition on some physiological processes in higher plants-growth, photosynthesis, photorespiration, and water relations. Plant Biol..

[27] Zhang Y., Duan Y., Shen Q. (2004). Screening of physiological indices for response of rice to nitrate. Acta Pedol. Sin..

[28] Song N., Guo S.W., Shen Q. (2007). Effects of different nitrogen forms and water stress on water absorption, photosynthesis, and growth of rice seedlings. Acta Bot. Sin..

[29] Imsande J. (1986). Nitrate-ammonium ratio required for pH homeostasis in hydroponically grown soybean. J. Exp. Bot..

[30] Li K., Guo Y.Q., Liu C.N., Lu X., Liao H. (2014). Effects of ammonium-nitrate ratio on growth and nodulation in soybean. Chin. J. Oil Crop Sci..

[31] Chen L., Zhu Y.L., Yang L.F., Wang C. (2010). Effects of different nitrogen form ratios on growth, seed antioxidant enzyme activity, and reactive oxygen metabolism in vegetable soybean. J. Plant Nutr. Fert..

[32] Bernardo L.M., Clark R.B., Maranville J.W. (1984). Nitrate/ammonium ratio effects on nutrient solution pH, dry matter yield, and nitrogen uptake of sorghum. J. Plant Nutr..

[33] Tang G.J., Gao C.L., Jiang S.D., Zhang Y., Zhou X.X., Li Y.P., Xu T.Y. (2014). Effects of nitrogen forms and ratios on nitrogen use efficiency and quality of flue-cured tobacco. Hunan Agric. Sci..

[34] Liu S.L., Hua D.L., Jie X.L., Lei G.H., Zhang H.T., Liu F., Zhu J.F. (2010). Effects of different ammonium/nitrate ratios in nutrient solutions on tobacco mineral nutrient absorption and accumulation. Soil Bull..

[35] Matt P., Geiger M., Walch L.P., Engels C., Krapp A., Stitt M. (2001). Elevated carbon dioxide increases nitrate uptake and nitrate reductase activity when tobacco is growing on nitrate, but increases ammonium uptake and inhibits nitrate reductase activity when tobacco is growing on ammonium nitrate. Plant Cell Environ..

[36] Geiger M., Haake V., Ludewig F., Sonnewald U., Stitt M. (1999). The nitrate and ammonium nitrate supply have a major influence on the response of photosynthesis, carbon metabolism, nitrogen metabolism and growth to elevated carbon dioxide in tobacco. Plant Cell Environ..

[37] Tabatabaei J.S., Yusefi M., Hajiloo J. (2008). Effects of shading and NO_3_^−^: NH_4_^+^ ratio on the yield, quality and N metabolism in strawberry. Sci. Hortic..

[38] Taghavi T.S., Babalar M., Ebadi A., Ebrahimzadeh H., Asgari M.A. (2004). Effects of nitrate to ammonium ratio on yield and nitrogen metabolism of strawberry (*Fragaria xananassa* cv. selva). Int. J. Agric. Biol..

[39] Francesco S., Antonio E., Angelo S., Pietro S. (2004). Influence of nitrogen form on yield and nitrate content of subirrigated early potato. J. Sci. Food Agric..

[40] Dobránszki J., Tábori K.M. (2010). Influence of nitrogen supply of potato plantlets on in vitro tuberization pattern under inductive and non-inductive conditions. Potato Res..

[41] Lu Y.L. (2009). Molecular Physiological Mechanisms of Ammonium-Nitrate Nutrition Affecting Tomato Seedling Growth and Nitrogen Metabolism.

[42] Li W., Wang Y., Okamoto M., Crawford N.M., Siddiqi M.Y., Glass A.D. (2007). Dissection of the *AtNRT2.1*:*AtNRT2.2*inducible high-affinity nitrate transporter gene cluster. Plant Physiol..

[43] Borrero C., Trillas M.I., Delgado A., Avilés M. (2012). Effect of ammonium/nitrate ratio in nutrient solution on control of Fusarium wilt of tomato by Trichoderma asperellum T34. Plant Pathol..

[44] Liu G., Du Q., Li J. (2017). Interactive effects of nitrate-ammonium ratios and temperatures on growth, photosynthesis, and nitrogen metabolism of tomato seedlings. Sci. Hortic..

[45] Flores P., Carvajal M., Cerdá A., Martínez V. (2001). Salinity and ammonium/nitrate interactions on tomato plant development, nutrition, and metabolites. J. Plant Nutr..

[46] Hu L., Liao W., Dawuda M., Yu J., Lv J. (2017). Appropriate NH_4_^+^: NO_3_^−^ ratio improves low light tolerance of mini Chinese cabbage seedlings. BMC Plant Biol..

[47] Chen W., Luo J., Shen Q. (2005). Effect of NH_4_^+^-N/NO_3_^−^-N ratios on growth and some physiological parameters of Chinese cabbage cultivars. Pedosphere.

[48] Bao L., Dong J.L., Li X., Duan Z.Q. (2016). Effects of elevated CO_2_ concentration and nitrogen supply on the photosynthetic pigments of cucumber leaves. Soil.

[49] Wang J.F., Dong C.X., Shen Q. (2007). Effects of different nitrogen forms on the content of free amino acids and related enzyme activities in spinach. J. Plant Nutr. Fertil..

[50] Zhang Y., Xu X., Lin X., Zhang Y., Du S., Li G. (2006). Effects of nitrogen forms on nitrate and oxalate accumulation in the edible part of spinach. J. Plant Nutr. Fert..

[51] Yang Y., Zheng Q.L., Pei C.G., Zhai H. (2010). Effects of different nitrate-ammonium ratios on growth and nitrogen nutrition in Chardonnay grape seedlings. J. Plant Nutr. Fert..

[52] Lv W.X., Hui Z.M. (2012). Effects of different nitrogen forms on the fruit quality of “Cabernet Sauvignon” grape. North Hortic..

[53] Gamiely S., Randle W., Mills H., Smittle D., Banna G. (1991). Onion plant growth, bulb quality, and water uptake following ammonium and nitrate nutrition. Hortscience.

[54] King J., Gay A., Sylvester-Bradley R., Bingham I., Foulkes J., Gregory P., Robinson D. (2003). Modelling cereal root systems for water and nitrogen capture: Towards an economic optimum. Ann. Bot..

[55] Mi G., Chen F., Chun L., Guo Y.F., Tian Q., Zhang F. (2007). Biological characteristics of nitrogen efficient maize genotypes. J. Plant Nutr. Fert..

[56] Mi G., Chen F., Wu Q., Lai N., Yuan L., Zhang F. (2010). Ideotype root architecture for efficient nitrogen acquisition by maize in intensive cropping systems. Sci. China (Life Sci.).

[57] Lynch J.P. (2013). Steep, cheap and deep: An ideotype to optimize water and N acquisition by maize root systems. Ann. Bot..

[58] Ren W., Zhao L., Liang J., Wang L., Chen L., Li P., Liu Z., Li X., Zhang Z., Li J. (2022). Genome-wide dissection of changes in maize root system architecture during modern breeding. Nat. Plants.

[59] Mi G., Chen F., Yuan L., Zhang F. (2016). Ideotype root system architecture for maize to achieve high yield and resource use efficiency in intensive cropping systems. Adv. Agron..

[60] Plett D., Toubia J., Garnett T., Tester M., Kaiser B.N., Baumann U. (2010). Dichotomy in the *NRT* gene families of dicots and grass species. PLoS ONE.

[61] Gu R., Duan F., An X., Zhang F., Von Wirén N., Yuan L. (2013). Characterization of *AMT*-mediated high-affinity ammonium uptake in roots of maize (*Zea mays* L.). Plant Cell Physiol..

[62] Hui J., Liu Z., Duan F., Zhao Y., Li X., An X., Wu X., Yuan L. (2022). Ammonium-dependent regulation of ammonium transporter *ZmAMT1s* expression conferred by glutamine levels in roots of maize. J. Integr. Agric..

[63] Kronzucker H.J., Glass A.D., Yaeesh S.M. (1999). Inhibition of nitrate uptake by ammonium in barley. Analysis of component fluxes. Plant Physiol..

[64] Dong C., Lu Y., Zhu Y., Zhou Y., Xu Y., Shen Q. (2012). Effect of homogeneous and heterogeneous supply of nitrate and ammonium on nitrogen uptake and distribution in tomato seedlings. Plant Growth Regul..

[65] Feil B. (1994). Growth and ammonium: Nitrate uptake ratio of spring wheat cultivars under a homogeneous and a spatially separated supply of ammonium and nitrate. J. Plant Nutr..

[66] Bar-Tal A., Aloni B., Karni L., Rosenberg R. (2001). Nitrogen nutrition of greenhouse pepper. II. Effects of nitrogen concentration and NO_3_^−^: NH_4_^+^ ratio on growth, transpiration, and nutrient uptake. HortScience.

[67] Kronzucker H.J., Siddiqi M.Y., Glass A.D., Kirk G.J. (1999). Nitrate-ammonium synergism in rice. A subcellular flux analysis. Plant Physiol..

[68] Cerezo M., Tillard P., Muños S., Daniel-Vedele F., Gojon A. (2001). Major alterations of the regulation of root NO_3_^−^ uptake are associated with the mutation of *Nrt2.1* and *Nrt2.2* genes in Arabidopsis. Plant Physiol..

[69] Babourina O., Voltchanskii K., Mcgann B., Newman I., Rengel Z. (2007). Nitrate supply affects ammonium transport in canola roots. J. Exp. Bot..

[70] Wirth J., Chopin F., Santoni V., Viennois G., Tillard P., Krapp A., Lejay L., Daniel-Vedele F., Gojon A. (2007). Regulation of root nitrate uptake at the *NRT2.1* protein level in Arabidopsis thaliana. J. Biol. Chem..

[71] Luo J., Qin J., He F., Li H., Liu T., Polle A., Peng C., Luo Z. (2013). Net fluxes of ammonium and nitrate in association with H+ fluxes in fine roots of Populus popularis. Planta.

[72] Flynn K.J., Fasham M.J.R., Hipkin C.R. (1997). Modelling the interactions between ammonium and nitrate uptake in marine phytoplankton. Philos. Trans. R. Soc. B: Biol. Sci..

[73] Nazoa P., Vidmar J.J., Tranbarger T.J., Mouline K., Damiani I., Tillard P., Zhuo D., Glass A.D., Touraine B. (2003). Regulation of the nitrate transporter gene *AtNRT2.1* in Arabidopsis thaliana: Responses to nitrate, amino acids and developmental stage. Plant Mol. Biol..

[74] Zhuo D., Okamoto M., Vidmar J.J., Glass A.D. (1999). Regulation of a putative high-affinity nitrate transporter *(Nrt2;1At)* in roots of Arabidopsis thaliana. Plant J..

[75] Zhong Y., Yan W., Chen J., Shangguan Z. (2014). Net ammonium and nitrate fluxes in wheat roots under different environmental conditions as assessed by scanning ion-selective electrode technique. Sci. Rep..

[76] Lanquar V., Loqué D., Hörmann F., Yuan L., Bohner A., Engelsberger W., Lalonde S., Schulze W., Von W., Frommer W. (2010). Feedback inhibition of ammonium uptake by a phospho-dependent allosteric mechanism in Arabidopsis. Plant Signal Behav..

[77] Hachiya T., Sakakibara H. (2017). Interactions between nitrate and ammonium in their uptake, allocation, assimilation, and signaling in plants. J. Exp. Bot..

[78] Coskun D., Britto D., Li M., Becker A., Kronzucker H. (2013). Rapid ammonia gas transport accounts for futile transmembrane cycling under NH_3_^−^/NH_4_^+^ toxicity in plant roots. Plant Physiol..

[79] Li G., Tillard P., Gojon A., Maurel C. (2016). Dual regulation of root hydraulic conductivity and plasma membrane aquaporins by plant nitrate accumulation and high-affinity nitrate transporter *NRT2.1*. Plant Cell Physiol..

[80] Lynch J.P. (2019). Root phenotypes for improved nutrient capture: An underexploited opportunity for global agriculture. New Phytol..

[81] Lynch J.P. (2018). Rightsizing root phenotypes for drought resistance. J. Exp. Bot..

[82] Uga Y., Sugimoto K., Ogawa S., Rane J., Ishitani M., Hara N., Kitomi Y., Inukai Y., Ono K., Kanno N. (2013). Control of root system architecture by DEEPER ROOTING 1 increases rice yield under drought conditions. Nat. Genet..

[83] Liu Y., Lai N., Gao K., Chen F., Yuan L., Mi G. (2013). Ammonium inhibits primary root growth by reducing the length of meristem and elongation zone and decreasing elemental expansion rate in the root apex in Arabidopsis thaliana. PLoS ONE.

[84] Giehl R.F.H., von Wirén N. (2014). Root nutrient foraging. Plant Physiol..

[85] Jia Z., von Wirén N. (2020). Signaling pathways underlying nitrogen-dependent changes in root system architecture: From model to crop species. J. Exp. Bot..

[86] Jia Z., Giehl R.F.H., von Wirén N. (2022). Nutrient-hormone relations: Driving root plasticity in plants. Mol. Plant..

[87] Yu P., Hochholdinger F., Li C. (2019). Plasticity of lateral root branching in maize. Front. Plant Sci..

[88] Zhang H., Jennings A., Barlow P., Forde B. (1999). Dual pathways for regulation of root branching by nitrate. Proc. Natl. Acad. Sci. USA.

[89] Zhang H., Rong H., Pilbeam D. (2007). Signalling mechanisms underlying the morphological responses of the root system to nitrogen in Arabidopsis thaliana. J. Exp. Bot..

[90] Lima J.E., Kojima S., Takahashi H., von Wirén N. (2010). Ammonium triggers lateral root branching in Arabidopsis in an AMMONIUM TRANSPORTER1;3-dependent manner. Plant Cell.

[91] Meier M., Liu Y., Lay-Pruitt K.S., Takahashi H., von Wirén N. (2020). Auxin-mediated root branching is determined by the form of available nitrogen. Nat. Plants.

[92] Oaks A., Poulle M., Goodfellow V., Cass L., Deising H. (1988). The role of nitrate and ammonium ions and light on the induction of nitrate reductase in maize leaves. Plant Physiol..

[93] Foyer C.H., Noctor G. (2006). Photosynthetic Nitrogen Assimilation and Associated Carbon and Respiratory Metabolism.

[94] Lewis C., Noctor G., Causton D., Foyer C. (2000). Regulation of assimilate partitioning in leaves. Funct. Plant Biol..

[95] Golvano M.P., Felipe M.R., Cintas A.M. (1982). Influence of nitrogen sources on chloroplast development in wheat seedlings. Physiol. Plant..

[96] Ikeda M., Kusano T., Koga N. (2004). Carbon skeletons for amide synthesis during ammonium nutrition in tomato and wheat roots. Soil Sci. Plant Nutr..

[97] Horchani F., Hajri R., Aschi-Smiti S. (2010). Effect of ammonium or nitrate nutrition on photosynthesis, growth, and nitrogen assimilation in tomato plants. J. Plant Nutr. Soil Sci..

[98] Høgh-Jensen H., Schjoerring J.K. (1997). Interactions between white clover and ryegrass under contrasting nitrogen availability: N_2_ fixation, N fertilizer recovery, N transfer and water use efficiency. Plant Soil.

[99] Raab T.K., Terry N. (1994). Nitrogen source regulation of growth and photosynthesis in *Beta vulgaris* L.. Plant Physiol..

[100] Hecht U., Mohr H. (1990). Factors controlling nitrate and ammonium accumulation in mustard (*Sinapis alba*) seedlings. Physiol. Plant..

[101] Claussen W., Lenz F. (1999). Effect of ammonium or nitrate nutrition on netphotosynthesis, growth, and activity of the enzymes nitrate reductase and glutamine synthetase in blueberry, raspberry and strawberry. Plant Soil..

[102] Arias-Baldrich C., Osa C.D.L., Bosch N., Ruiz-Ballesta I., Monreal J.A., García-Mauriño S. (2017). Enzymatic activity, gene expression and posttranslational modifications of photosynthetic and nonphotosynthetic phosphoenolpyruvate carboxylase in ammonium-stressed sorghum plants. J. Plant Physiol..

[103] Alencar V., Lobo A., Carvalho F., Silveira J. (2019). High ammonium supply impairs photosynthetic efficiency in rice exposed to excess light. Photosynth. Res..

[104] Pasqualini S., Ederli L., Piccioni C., Batini P., Bellucci M., Arcioni S., Antonielli M. (2001). Metabolic regulation and gene expression of root phosphoenolpyruvate carboxylase by different nitrogen sources. Plant Cell Environ..

[105] Hachiya T., Watanabe C., Fujimoto M., Ishikawa T., Takahara K., Kawaiyamada M., Uchimiya H., Uesono Y., Terashima I., Noguchi K. (2012). Nitrate addition alleviates ammonium toxicity without lessening ammonium accumulation, organic acid depletion and inorganic cation depletion in Arabidopsis thaliana shoots. Plant Cell Physiol..

[106] Masakapalli S.K., Kruger N.J., Ratcliffe R.G. (2013). The metabolic flux phenotype of heterotrophic Arabidopsis cells reveals a complex response to changes in nitrogen supply. Plant J..

[107] Sato S., Yanagisawa S. (2014). Characterization of metabolic states of Arabidopsis thaliana under diverse carbon and nitrogen nutrient conditions via targeted metabolomic analysis. Plant Cell Physiol..

[108] Lea P.J., Morot-Gaudry J.F. (2001). Plant Nitrogen.

[109] Masumoto C., Miyazawa S.I., Ohkawa H., Fukuda T., Taniguchi Y., Murayama S., Kusano M., Saito K., Fukayama H., Miyao M. (2010). Phosphoenolpyruvate carboxylase intrinsically located in the chloroplast of rice plays a crucial role in ammonium assimilation. Proc. Natl. Acad. Sci. USA.

[110] Shi J., Yi K., Liu Y., Xie L., Zhou Z., Chen Y., Hu Z., Zheng T., Liu R., Chen Y. (2015). Phosphoenolpyruvate carboxylase in Arabidopsis leaves plays a crucial role in carbon and nitrogen metabolism. Plant Physiol..

[111] Ruan Y.L. (2014). Sucrose metabolism: Gateway to diverse carbon use and sugar signaling. Annu. Rev. Plant Biol..

[112] Lawlor D.W., Paul M.J. (2014). Source/sink interactions underpin crop yield: The case for trehalose 6-phosphate/SnRK1 in improvement of wheat. Front. Plant Sci..

[113] Yu S., Lo S., Ho T.D. (2015). Source-sink communication: Regulated by hormone, nutrient, and stress cross-signaling. Trends Plant Sci..

[114] Martin T., Oswald O., Graham I.A. (2002). Arabidopsis seedling growth, storage lipid mobilization, and photosynthetic gene expression are regulated by carbon: Nitrogen availability. Plant Physiol..

[115] Moore B., Zhou L., Rolland F., Hall Q., Cheng W., Liu Y., Hwang I., Jones T., Sheen J. (2003). Role of the Arabidopsis glucose sensor HXK1 in nutrient, light, and hormonal signaling. Science.

[116] Dai T., Cao W., Sun C., Jiang D., Jing Q. (2003). Effect of enhanced ammonium nutrition on photosynthesis and nitrate reductase and glutamine synthetase activities of winter wheat. Chin. J. Appl. Ecol..

[117] Neales T.F., Incoll L.D. (1968). The control of leaf photosynthesis rate by the level of assimilate concentration in the leaf: A review of the hypothesis. Bot. Rev..

[118] Figueroa C.M., Lunn J.E. (2016). A tale of two sugars: Trehalose 6-phosphate and sucrose. Plant Physiol..

[119] Yadav U.P., Ivakov A., Feil R., Duan G.Y., Walther D., Giavalisco P., Piques M., Carillo P., Hubberten H., Stitt M. (2014). The sucrose-trehalose 6-phosphate (Tre6P) nexus: Specificity and mechanisms of sucrose signalling by Tre6P. J. Exp. Bot..

[120] Griffiths C., Sagar R., Geng Y., Primavesi L., Patel M., Passarelli M., Gilmore I., Steven R., Bunch J., Paul M. (2016). Chemical intervention in plant sugar signalling increases yield and resilience. Nature.

[121] Petrásek J., Friml J. (2009). Auxin transport routes in plant development. Development.

[122] Fu Y., Yang X., Zhang Z., Yuan S. (2022). Synergistic effects of nitrogen metabolites on auxin regulating plant growth and development. Front. Plant Sci..

[123] Reinhardt D., Mandel T., Kuhlemeier C. (2000). Auxin regulates the initia-tion and radial position of plant lateral organs. Plant Cell..

[124] Sieburth L.E. (1999). Auxin is required for leaf vein pattern in Arabidopsis. Plant Physiol..

[125] Keller C.P. (2017). Leaf expansion in Phaseolus: Transient auxin-induced growth increase. Physiol. Plant..

[126] Avery G.S., Pottorf L. (1945). Auxin and nitrogen relationships in green plants. Am. J. Bot..

[127] Tian Q., Chen F., Liu J., Zhang F., Mi G. (2008). Inhibition of maize root growth by high nitrate supply is correlated with reduced IAA levels in roots. J. Plant Physiol..

[128] Caba J.M., Centeno M.L., Fernández B., Gresshoff P.M., Ligero F. (2000). Inoculation and nitrate alter phytohormone levels in soybean roots: Differences between a supernodulating mutant and the wild type. Planta.

[129] Tamaki V., Mercier H. (2007). Cytokinins and auxin communicate nitrogen availability as long-distance signal molecules in pineapple (*Ananas comosus*). J. Plant Physiol..

[130] Ma W., Li J., Qu B., He X., Zhao X., Li B., Fu X., Tong Y. (2014). Auxin bio-synthetic gene *TAR2* is involved in low nitrogen-mediated reprogramming of root architecture in Arabidopsis. Plant J..

[131] Revsbech N.P., Pedersen O., Reichardt W., Briones A. (1999). Microsensor analysis of oxygen and pH in the rice rhizosphere under field and laboratory conditions. Biol. Fertil. Soils..

[132] Reed R.C., Brady S.R., Muday G.K. (1998). Inhibition of auxin movement from the shoot into the root inhibits lateral root development in Arabidopsis. Plant Physiol..

[133] Meng L., Dong J., Wang S., Song K., Ling A., Yang J., Xiao Z., Li W., Song W., Liang H. (2018). Differential responses of root growth to nutrition with different ammonium/nitrate ratios involve auxin distribution in two tobacco cultivars. J. Integr. Agric..

[134] Wang B., Shen Q. (2012). Effects of ammonium on the root architecture and nitrate uptake kinetics of two typical lettuce genotypes grown in hydroponic systems. J. Plant Nutr..

[135] Song W., Makeen K., Wang D., Zhang C., Xu Y., Zhao H., Tu E., Zhang Y., Shen Q., Xu G. (2011). Nitrate supply affects root growth differentially in two rice cultivars differing in nitrogen use efficiency. Plant Soil..

[136] Sun H., Feng F., Liu J., Zhao Q. (2019). Nitric Oxide Affects Rice Root Growth by Regulating Auxin Transport Under Nitrate Supply. Front. Plant Sci..

[137] Walch L.P., Neumann G., Bangerth F., Engels C. (2000). Rapid effects of nitrogen form on leaf morphogenesis in tobacco. J. Exp. Bot..

[138] Wang G., Li C., Zhang F. (2003). Effects of different nitrogen forms and combination with foliar spraying with 6-benzylaminopurine on growth, transpiration, and water and potassium uptake and flow in tobacco. Plant Soil..

[139] Martins-Loução M.A., Dias T., Cruz C. (2022). Integrating ecological principles for addressing plant production security and move beyond the dichotomy ‘good or bad’ for nitrogen inputs choice. Agronomy.

[140] Chen J., Li J., Li W., Li P., Zhu R., Zhong Y., Zhang W., Li T. (2024). The optimal ammonium–nitrate ratio for various crops: A meta-analysis. Field Crops Res..

